# Profiling circRNA and miRNA of radiation-induced esophageal injury in a rat model

**DOI:** 10.1038/s41598-018-33038-1

**Published:** 2018-10-02

**Authors:** Judong Luo, Changsong Zhang, Qiang Zhan, Fangmei An, Wenyu Zhu, Hua Jiang, Changsheng Ma

**Affiliations:** 1 Department of Radiation Oncology, Shandong Cancer Hospital and Institute, Shandong Cancer Hospital Affiliated to Shandong University, Shandong Academy of Medical Sciences, Jinan, China; 2 Medical college of Shandong University, Jinan, China; 3 Department of Oncology, The Affiliated Changzhou No. 2 People’s Hospital of Nanjing Medical University, Changzhou, China; 4 Department of Oncology, Changzhou Tumor Hospital, Soochow University, Changzhou, China; 5 Department of Gastroenterology, The Affiliated Wuxi People’s Hospital of Nanjing Medical University, Wuxi, China

## Abstract

Evidence has also shown that micro ribonucleic acid (miRNA) plays an important role in many cellular processes. However, it is unclear how ionizing radiation causes the miRNA and circular ribonucleic acid (circRNA) expression levels to change and how this change relates to esophageal injury. We analyzed RNA Sequencing (RNA-seq) data from normal esophageal tissue and irradiated esophageal tissues and used computational approaches to identify and characterize differentially expressed miRNAs and circRNAs. We detected 27 miRNAs and 197 circRNAs that had significantly different expression levels after ionizing radiation treatment compared with normal control.Among the 27 miRNAs, 7 miRNAs were down-regulated, and the other 20 were up-regulated. Their target genes were found to be involved in responses to wound, lipid biosynthesis, cell proliferation, cell migration, chemokine activity, hairpin binding, and the cell membrane system. We also found 197 differentially expressed circRNAs in total, of which 87 were up-regulated and 110 were down-regulated. Notably, we found that differentially expressed circRNAs were enriched in cell differentiation, epithelial cell migration, striatum development, protein binding, extracellular exosome, and focal adhesion functions. Of the related processes, sphingolipid metabolism was notable. Many of the differentially expressed circRNAs were involved in sphingolipid metabolism pathways. Cells responded to ionizing radiation (IR) using multiple pathways, which led to sphingolipid metabolism and other immune responses, ultimately leading to esophageal injury.IR-induced esophageal injury is worth studying, especially the dynamic network of circRNA and miRNA. By knowing the regulatory details of related pathways, radiation-related esophageal injury can be prevented, and the efficiency of radiation therapy can be enhanced.

## Introduction

Radiation therapy, using high dose of radiation to kill tumor cells, is one of the most common and important treatment for cancer. Since esophageal epithelial cells are extremely sensitive to ionizing radiation, during radiation therapy, they are vulnerable to the damage caused by high-energy radiation^1^. Radiation-induced esophageal injury commonly occurs in the patients who received radiation therapy for the carcinomas in the region of neck, chest, or mediastinum^2^. Radiation-induced acute esophagitis is also the cause for suspension or failure of radiotherapy^3,4^. In addition, ionizing radiation was reported to have the potential to increase the risk of esophageal cancer in patients receiving radiotherapy for primary carcinomas located in the head and neck, breast and mediastinal regions^5–10^.

The mechanisms of radiation-induced cell damage can be divided into two categories: direct and indirect action. Direct action means that radiation acts directly on cellular deoxyribonucleic acid (DNA), causing the breakage of DNA. Indirect action means radiation causes the ionization of water molecules to produce free radicals, which act on important molecules in cells, such as nucleic acids, lipids and proteins, leading to cellular function disorders or even cell death^11^. Despite of that, ionizing radiation could induce mutations in oncogenes such as Minor Allele Frequency (MAF), tumor suppressor genes such as p53, or DNA repair genes such as Asynchronous Transfer Mode (ATM)^12–14^. Ionizing radiation could also lead to the mutation of genes that are involved in intercellular interaction and inflammation^15,16^, and enhance the migration and invasion of esophageal epithelial cells via stromal-derived hepatocyte growth factors^17^. On the contrast, ionizing radiation induce cells to initiate the transcription and translation of multiple proteins and noncoding RNAs, forming a complex radiation stress-induced regulation network, to defend themselves from the damage caused by radiation^18–20^.

Although multiple mechanisms have been proposed for the development of esophagitis, the underlying molecular signaling events remain uncertain. Circular RNAs (CircRNAs) are a unique class of non-coding RNA. Different from linear RNA, circRNAs are structured by a covalently closed loop; therefore they do not contain either 5′-3′ polarity or polyadenylated tail^21^. It has been well demonstrated that circRNAs are involved in the regulation of multiple biological processes^22–24^, including cancer development^25,26^, progression^27^ as well as suppression^28^. MicroRNAs (miRNAs) are small, highly conserved non-coding RNA molecules involved in the regulation of gene expression. CircRNAs were reported to have the ability to act as the miRNAs sponges, which inhibit miRNAs access to their target mRNAs by competing for the same binding site of miRNAs, thereby suppressing the target gene of the respective miRNAs^29^. To date, there is no reports focusing on pathways of intercellular communication and circRNA-miRNA-mRNA network in the radiation-induced injury in esophageal tissues.

RNA-sequencing (RNA-seq), together with the improvements in experimental technologies, facilitates the acquisition of more accurate results. The RNA-seq method correlates well with the microarray method and can detect more genes than microarrays^30^. In this study, RNA-seq was used to investigate ionizing radiation-responsive genes in normal esophageal tissue. Also, the genome-wide expression in esophageal tissues was compared between normal and the radiation therapy group, considering the functional categories of differentially expressed miRNAs and circRNAs. Gene ontology (GO) was used to analyze differentially expressed miRNAs and circRNAs. Together, these analyses provided information for research on radiotherapy that might have a side effect on the esophagus.

## Materials and Methods

### Animals and treatments

Animal welfare and experimental procedures were carried out in accordance with the Guide for the Care and Use of Laboratory Animals (the Shanghai SLAC Laboratory Animal of China, 2015), and were approved by the animal ethics com-mittee of Shandong Cancer Hospital and Institute (No. of Licence 201606021). Male Sprague-Dawley (SD) rats (4 weeks old) were purchased from the Shanghai SLAC Laboratory Animal Co., Ltd. (Shanghai, China). The animals were housed with a 12-h light/dark cycle with food and water ad libitum. After anesthetized with an intraperitoneal injection of ketamine (75 mg/kg) and xylazine (10 mg/kg), the rats were fixed with adhesive tape on a plate to avoid movement during radiation exposure. A plate of lead was used to localize the radiation area (3 cm × 4 cm). The esophageal area was subjected to a single dose of 0, 5 or 20 Gy irradiation at the rate of 2 Gy/min (5 rates per treatment group) by using a 6-MeV X-ray irradiation (Clinac 2100EX, Varian Medical Systems, Inc., CA). In the control group(n = 5), the rats were subjected to mock irradiation. The body was covered outside the irradiated area with lead skin both for experimental group and control group. Seven days after irradiation, the rats were sacrificed and the esophageal tissues were collected for analysis. All animal experiments were conducted according to legal regulations in China and carried out with the permission and under the regulation of the Institutional Animal Care and Use Committee of National Institute of Radiological Sciences.

### Hematoxylin and eosin (H&E) staining

Esophageal tissues were fixed in 4% paraformaldehyde overnight at 4 °C, and then embedded in paraffin. The 3 um thick sections were deparaffinized and stained with H&E. The morphology changes of esophageal tissues were observed after H&E staining.

### High-throughput RNA sequencing of circRNA

Total RNAs were extracted from esophageal tissues using Trizol reagent according to the manufacturer’s protocol (Invitrogen, CA, USA). Quantification of RNA was conducted using NanoDrop ND-2000C spectrophotometer (Thermo, CA, USA). The concentration of total RNA was measured by spectrophotometer. Only sample with a 260/280 ratio of ~2.0 were used for analysis. Quantification of circRNAs were performed by Shanghai Genergy Biotech (Shanghai, China). A high throughput RNA sequencing was conducted after removing ribosomal RNA and building a library. The Bowtie2 (http://bowtie-bio.sourceforge.net/bowtie2/manual.shtml) was used to align the reads against reference genomes. A back-splice algorithm was used to pick out the junctions of unmapped reads. Finally, further analysis was performed considering circRNAs as reference sequence. The expression level of circRNAs was quantified by “mapped back-splicing junction reads per million mapped reads” (RPM).

### High-throughput RNA sequencing of miRNAs

Total RNAs were separated by agarose gel electrophoresis. The RNAs that were smaller than 50 nt were enriched and ligated. The ligated RNAs were reverse-transcribed and amplified by polymerase chain reaction (PCR) using gene-specific primers. An Illumina Hiseq 2500 (Santiago, CA, USA) platform at the Shanghai Genergy Biotech (Shanghai, China) was used to perform rat sRNA libraries sequencing.

### Differentially expressed circRNA and miRNA analysis

The raw sequencing data were normalized by programming with R launguage. Transcript expression was calculated in fragments per kilobase of transcript per million fragments mapped (FPKM). We screened for known differentially expressed transcripts using the cuffdiff program. The level of expression was calculated using the cuffnorm program. A transcript was determined to be differentially expressed if the difference in expression levels had a p < 0.05, and those showing a ≥2-fold difference in the expression between the groups were screened. Cluster software was adopted to analyze differentially expressed circRNAs and miRNAs. Hierachical clustering (HCL) was used to further analyze the normalized expression level.

### LncRNA-miRNA, miRNA-mRNA, and circRNA-miRNA co-expression network

The Long non-coding RNA (lncRNA)-miRNA co-expression network was constructed based on normalized signal intensity of individual transcripts. Strongly related lncRNAs -miRNAs pairs, identified as having a Pearson’s correlation coefficient value of 0.8 or above and a p-value less than 0.05, were included in the co-expression network. As in the lncRNA-miRNA network, miRNA-mRNA and circRNA-miRNA co-expression networks were built.

### GO and pathway analysis

Functions analysis of circRNAs, miRNAs and their co-expressed genes were analyzed with the database for Annotation, Visualization and Intergrated Discovery (DAVID). GO functional annotation of co-expressed genes was performed to further predict the possible functions of circRNAs and miRNAs. Genes were divided into three subgroups according to their functions: biological process (BP), cellular component (CC) and molecular function (MF). The the Kyoto Encyclopedia of Genes and Genomes (KEGG) pathway analysis was also been performed in order to explore the biological pathways which the co-expressed genes were potentially to be involved in. Ven analysis was performed to further investigate the most common pathways in which differentially expressed or co-expressed genes are involved.

## Results

### Radiation induces damage to esophagus in rat models

**Figure 1:**
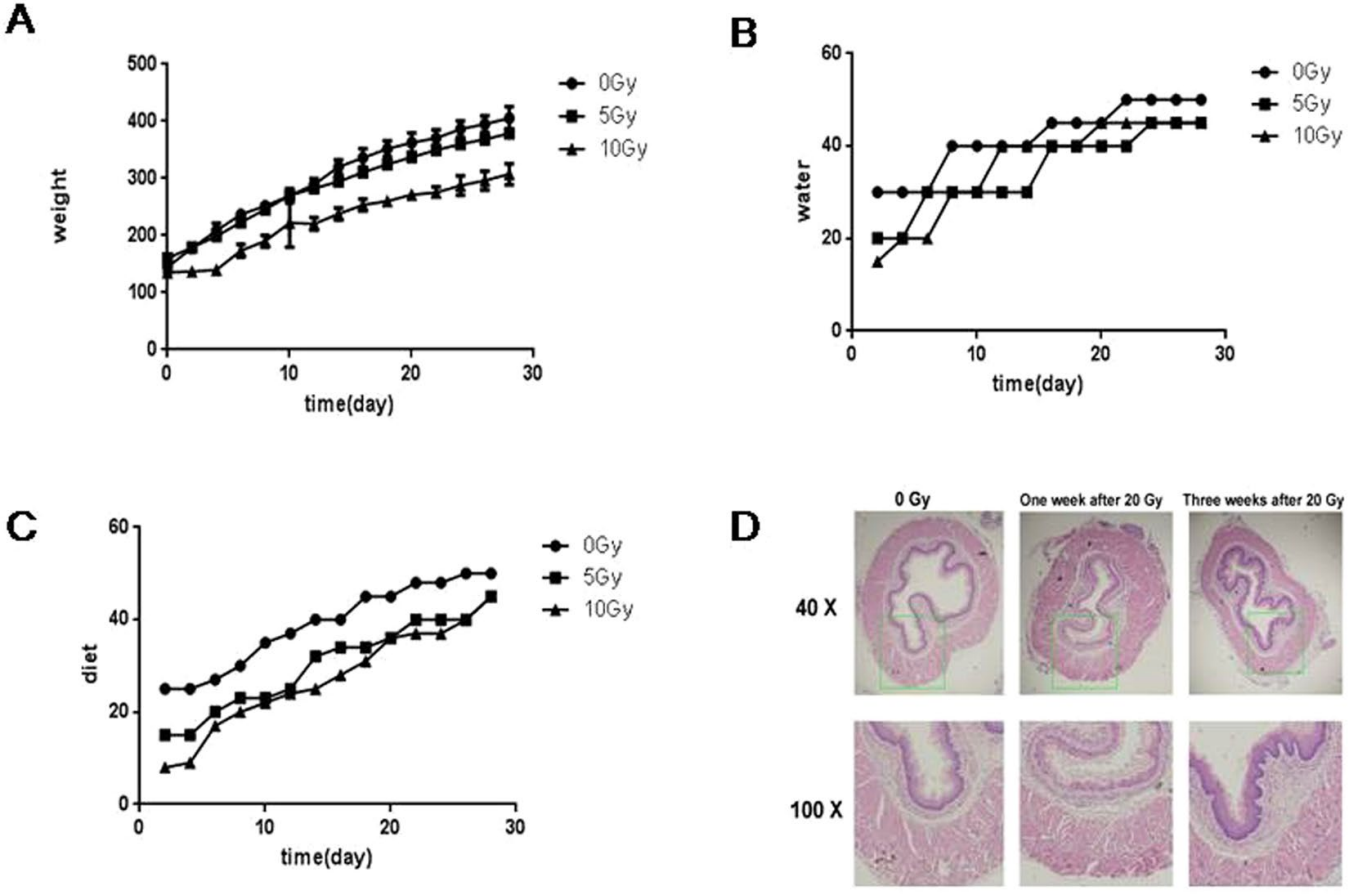
The effect of radiation on rat esophagus. A single dose of 0, 5 or 20 Gy irradiation was administered to the esophageal area. (**A**) Body weight of each group. (**B**) Water intake of each group. (**C**) Food intake of each group. (**D**) Representative H&E staining of rat esophagus after irradiation.

To investigate the effects of radiation on the rat esophagus, a single dose of 0, 5 or 20 Gy radiation was administered to the esophageal area of rats. Rats with 5 Gy irradiation showed similar body weight increases compared to the control group (0 Gy), whereas 20 Gy-irradiated rats showed a significant decrease in body weight (Fig. 1A). Rats with 5 and 20 Gy did not show differences in water intake (Fig. 1B), but there was a reduced dietary intake (Fig. 1C). HE staining showed that there was inflammatory cell infiltration and capillary telangiectasis in the esophagus of rats 7 days after irradiation (Fig. 1D). Three weeks after irradiation, esophageal hyperplasia and fibrosis emerged (Fig. 1D). The above results indicated that irradiation caused morphological and functional damage to the esophagus in rat models.

### Differentially expressed miRNA

**Figure 2:**
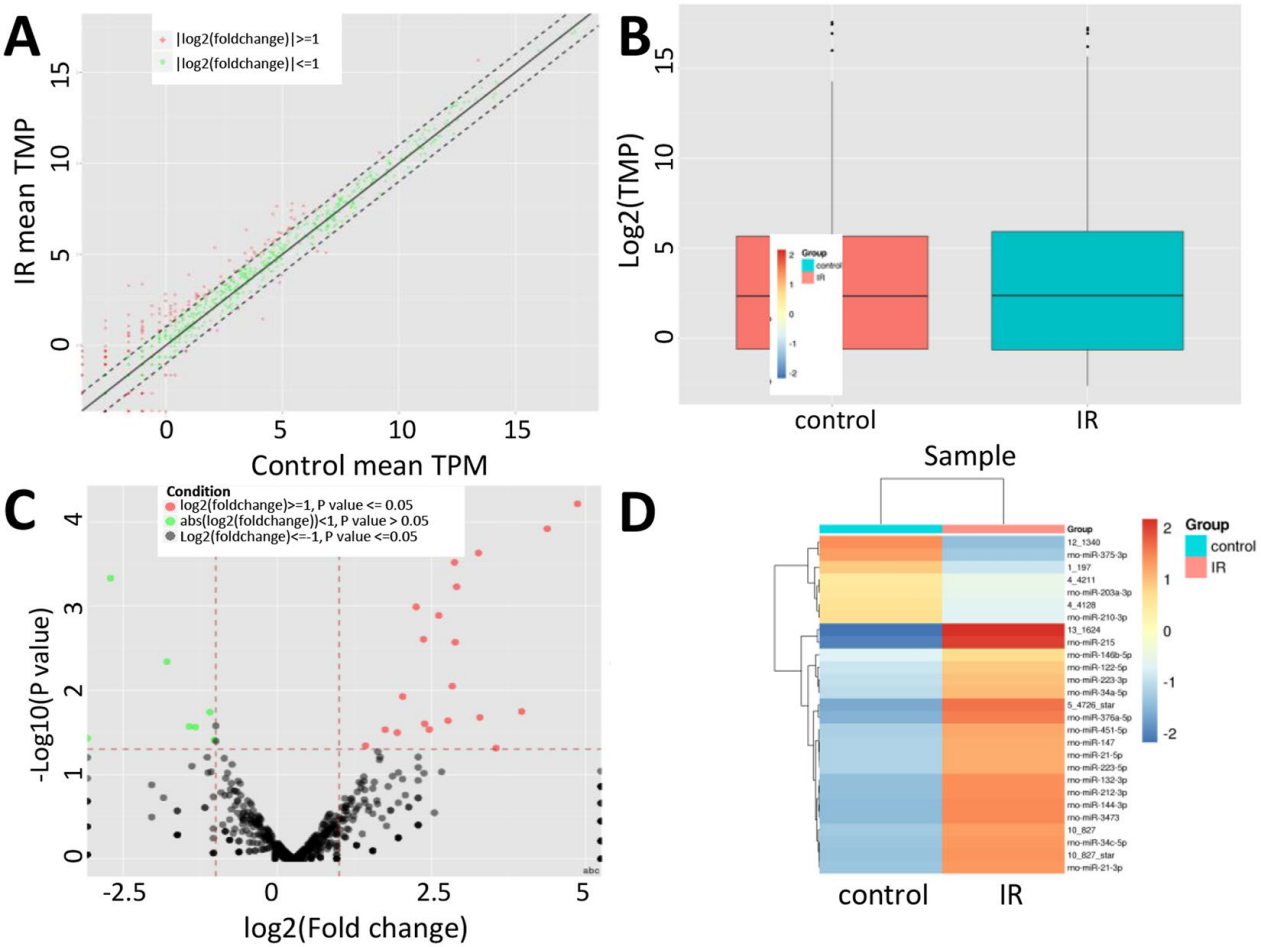
Identification of differentially expressed miRNAs. (**A**) Scatter plots assess the miRNA expression variation between the two compared groups. The miRNAs above the dotted line and below the bottom dotted line indicated more than two-fold changes between the two compared samples. (**B**) Box plot of log2 (FPKM) values across control and IR miRNAs. Control, normal esophageal tissue sample and IR, ionizing radiation treated sample. (**C**) Volcano plot of the differentially expressed miRNAs. The vertical lines correspond to 1.5-fold up and down, respectively, and the horizontal line represents P = 0.05. The green and red dots in the plot represent the differentially expressed miRNAs with statistical significance. (**D**) Heat map showing expression profiles of differentially expressed miRNAs.

In the present study, the transcriptional expression level was converted to a FPKM value using the Cufflink software. The differential expression of miRNA levels between the control and radiation-treated groups was evaluated by Cufflink and the dotted line indicated that the cutoff fold changed less than two (Fig. 2A). The box plot showed that the normalized fragments per kilobaseof exon per million fragments mapped (FPKM) densities for all genes were similar between control and radiation-treated esophageal tissues (Fig. 2B). The volcano plots show the differential expression of miRNA in two samples (Fig. 2C).

With the cutoff criteria of false discovery rates (FDRs) ≤ 0.05 and |log2 fold changes| ≥ 1.0, we identified 27 differentially expressed miRNAs between control and radiation-treated samples, including 20 up-regulated and 7 down-regulated genes (Fig. 2D and Table 1). The log2 (fold changes) of the differentially expressed genes in radiation-treated samples compared to controls ranged from −13.82 to 15.32. Since each miRNA can regulate multiple mRNAs^29^, we used TargetScan (http://www.targetscan.org/) to predict potential targets and analyze these mRNAs by KEGG. Pathways such as Wnt, TGF-beta and fatty acid elongation are significantly enriched.

**Table 1:** Differentially expressed miRNAs between control and irradiation treated samples.

MiRNAs	TPM(Mean)		Log2 Fold Change	P-value
	Control	IR		
**Down-regulated**				
rno-miR-375-3p	17.73	2.72	−2.71	0.00
1_197	116.56	33.72	−1.79	0.00
rno-miR-210-3p	29.22	10.87	−1.43	0.03
4_4128	90.45	35.96	−1.33	0.03
rno-miR-203a-3p	17582.21	8230.90	−1.09	0.02
4_4211	439.63	216.40	−1.02	0.04
12_1340	0.98	0.00	N/A	0.04
**Up-regulated**				
rno-miR-146b-5p	576.05	1549.01	1.43	0.05
rno-miR-122-5p	59.76	200.90	1.75	0.03
rno-miR-223-3p	6.57	25.25	1.94	0.03
rno-miR-34a-5p	41.70	170.53	2.03	0.01
rno-miR-21-5p	10818.19	51481.47	2.25	0.00
rno-miR-451-5p	42.52	219.76	2.37	0.00
rno-miR-223-5p	1.31	6.87	2.39	0.02
rno-miR-147	0.98	5.43	2.46	0.03
rno-miR-34c-5p	24.13	148.00	2.62	0.00
10_827	0.66	4.48	2.77	0.02
rno-miR-21-3p	0.98	7.03	2.84	0.01
rno-miR-144-3p	27.09	198.34	2.87	0.00
rno-miR-132-3p	1.97	14.54	2.88	0.00
rno-miR-3473	10.83	81.19	2.91	0.00
5_4726_star	4.27	40.91	3.26	0.00
rno-miR-212-3p	0.33	3.20	3.28	0.02
10_827_star	0.16	1.92	3.55	0.05
rno-miR-376a-5p	0.16	2.56	3.96	0.02
rno-miR-215	0.49	10.23	4.38	0.00
13_1624	0.33	9.59	4.87	0.00

TPM: Transcripts per million.

### Identification of differentially expressed circRNA

**Figure 3:**
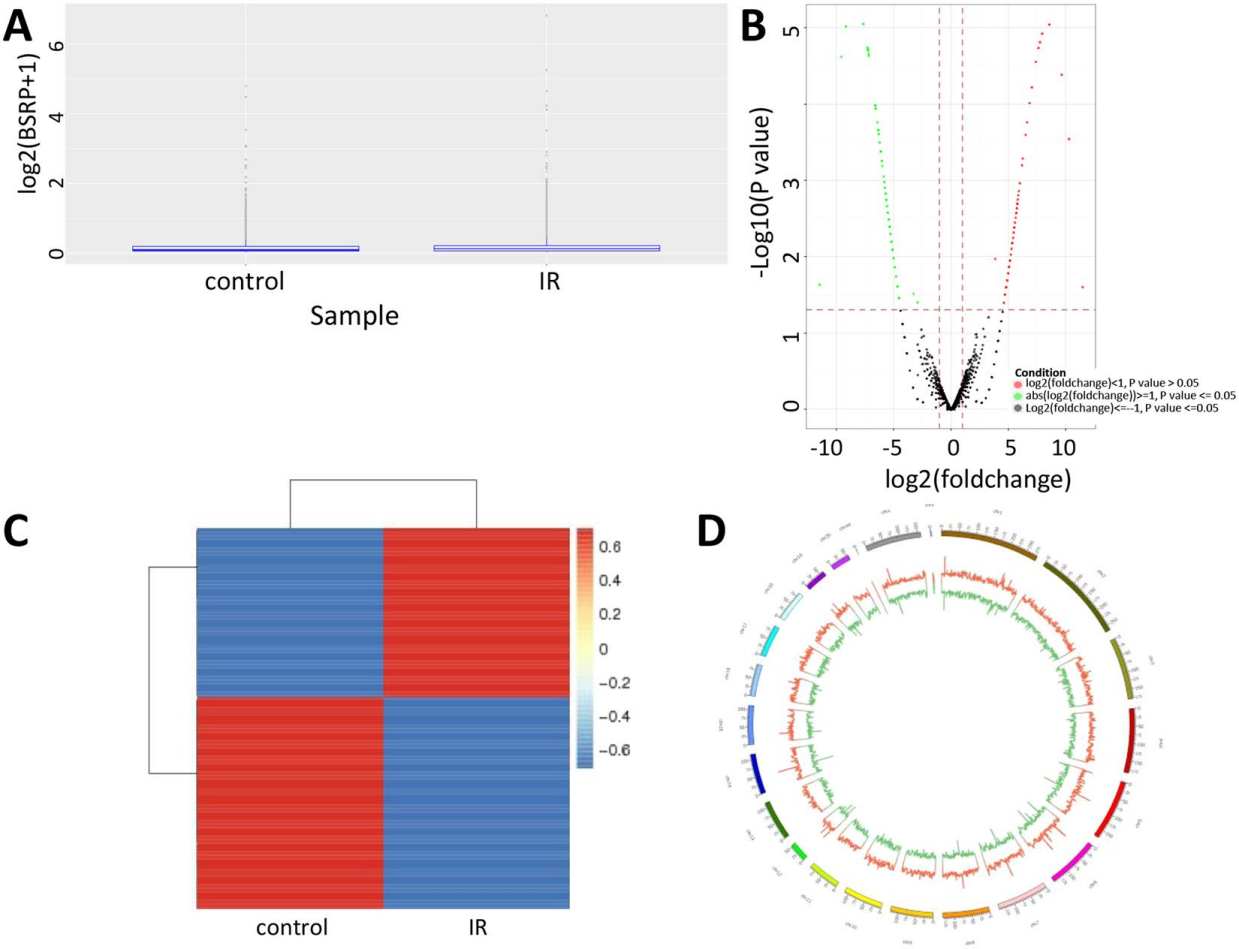
Identification of differentially expressed circRNAs. (**A**) Box plots show the distribution of circRNAs for the two compared samples. The distributions were nearly the same after normalization. (**B**) Volcano plot of the differentially expressed circRNAs. The vertical lines correspond to 1.5-fold up and down, respectively, and the horizontal line represents P = 0.05. The red and green dots in the plot represents the differentially expressed circRNAs with statistical significance. (**C**) Heat map showing expression profiles of differentially expressed circRNAs. (**D**) Circular plot showing differentially expressed circRNAs on human chromosomes. The increased or decreased circRNAs have been marked in red or blue bars, respectively.

To study the expression profile of circRNAs in radiation-induced esophageal injury, we compared the circRNA expression profiles in irradiated rats to normal controls using RNA-Seq analysis. After normalization, the distribution of log2 ratios among samples were nearly the same (Fig. 3A). Differentially expressed circRNAs with statistical significance between the groups were identified using volcano plot filtering (Fig. 3B). There are 197 differentially expressed circRNAs in total, with 110 down-regulated and 87 up-regulated. We chose 74 differentially expressed circRNAs, of which 37 were down-regulated and 37 were up-regulated, to present in the heat map (Fig. 3C). The top 20 differentially expressed circRNAs are listed in Table 2. The circRNAs are widely distributed in almost all the chromosomes, except for chromosome 21, chromosome 22 and chromosome Y (Fig. 3D).

**Table 2:** Top 20 differentially expressed cicrRNAs between control and irradiation treated samples.

CicrRNAs	Genes	Mean		Log2 Fold Change	P-value
		G1_base	G2_base		
**Down-regulated**					
10:53626328|53821461	N/A	2826.39	0.00	−11.47	0.02
7:143316920|143324536	ENSRNOG00000050420	764.74	0.00	−9.58	0.00
13:27159238|27192617	N/A	572.43	0.00	−9.16	0.00
2:122507139|122625548	ENSRNOG00000012734	201.25	0.00	−7.66	0.00
3:95413081|95413259	ENSRNOG00000013452	154.29	0.00	−7.28	0.00
7:118746211|118997203	N/A	152.05	0.00	−7.26	0.00
14:86470719|86603241	ENSRNOG00000005130	149.82	0.00	−7.24	0.00
7:118746529|118997669	N/A	149.82	0.00	−7.24	0.00
10:88033807|88033875	ENSRNOG00000014070	145.34	0.00	−7.19	0.00
19:15149359|15161377	ENSRNOG00000015519 ENSRNOG00000015438	143.11	0.00	−7.17	0.00
3:67719259|67746121	N/A	96.15	0.00	−6.60	0.00
6:43740210|43758721	N/A	96.15	0.00	−6.60	0.00
14:86479949|86612905	ENSRNOG00000005130	96.15	0.00	−6.60	0.00
7:118745796|118996133	N/A	96.15	0.00	−6.60	0.00
8:6203304|6349517	N/A	93.91	0.00	−6.57	0.00
7:118752999|119003711	N/A	93.91	0.00	−6.57	0.00
7:118766385|119016713	N/A	93.91	0.00	−6.57	0.00
18:35166777|35426253	N/A	84.97	0.00	−6.43	0.00
7:118764020|119013567	N/A	80.50	0.00	−6.35	0.00
6:135020549|135081711	ENSRNOG00000006178	80.50	0.00	−6.35	0.00
**Up-regulated**					
20:2663161|2676199	ENSRNOG00000047657	6.71	96.60	3.85	0.01
14:24107435|24110880	ENSRNOG00000002002	0.00	23.26	4.60	0.04
1:64033493|64101327	N/A	0.00	23.26	4.60	0.04
13:109781516|109820703	N/A	0.00	23.26	4.60	0.04
7:18416266|18417952	ENSRNOG00000008857	0.00	23.26	4.60	0.04
13:35579757|35608096	N/A	0.00	23.26	4.60	0.04
6:144189453|144275272	N/A	0.00	23.26	4.60	0.04
10:92474821|92476192	N/A	0.00	23.26	4.60	0.04
9:11134771|11135157	N/A	0.00	23.26	4.60	0.04
8:79330088|79338690	ENSRNOG00000058898	0.00	23.26	4.60	0.04
18:15480332|15494701	ENSRNOG00000015895	0.00	23.26	4.60	0.04
11:85892885|86255856	N/A	0.00	23.26	4.60	0.04
6:871525|872927	ENSRNOG00000004208	0.00	23.26	4.60	0.04
14:107594175|107594869	ENSRNOG00000009267	0.00	23.26	4.60	0.04
10:86987780|86991688	ENSRNOG00000027350	0.00	23.26	4.60	0.04
12:15075273|15098764	ENSRNOG00000001103	0.00	23.26	4.60	0.04
6:136059672|136081622	ENSRNOG00000010330	0.00	23.26	4.60	0.04
1:40433889|40459139	ENSRNOG00000016011	0.00	23.26	4.60	0.04
14:78071959|78234761	N/A	0.00	25.04	4.70	0.03
9:98239940|98247570	ENSRNOG00000019892	0.00	25.04	4.70	0.03

### Functional annotation of the differentially expressed miRNAs

**Figure 4:**
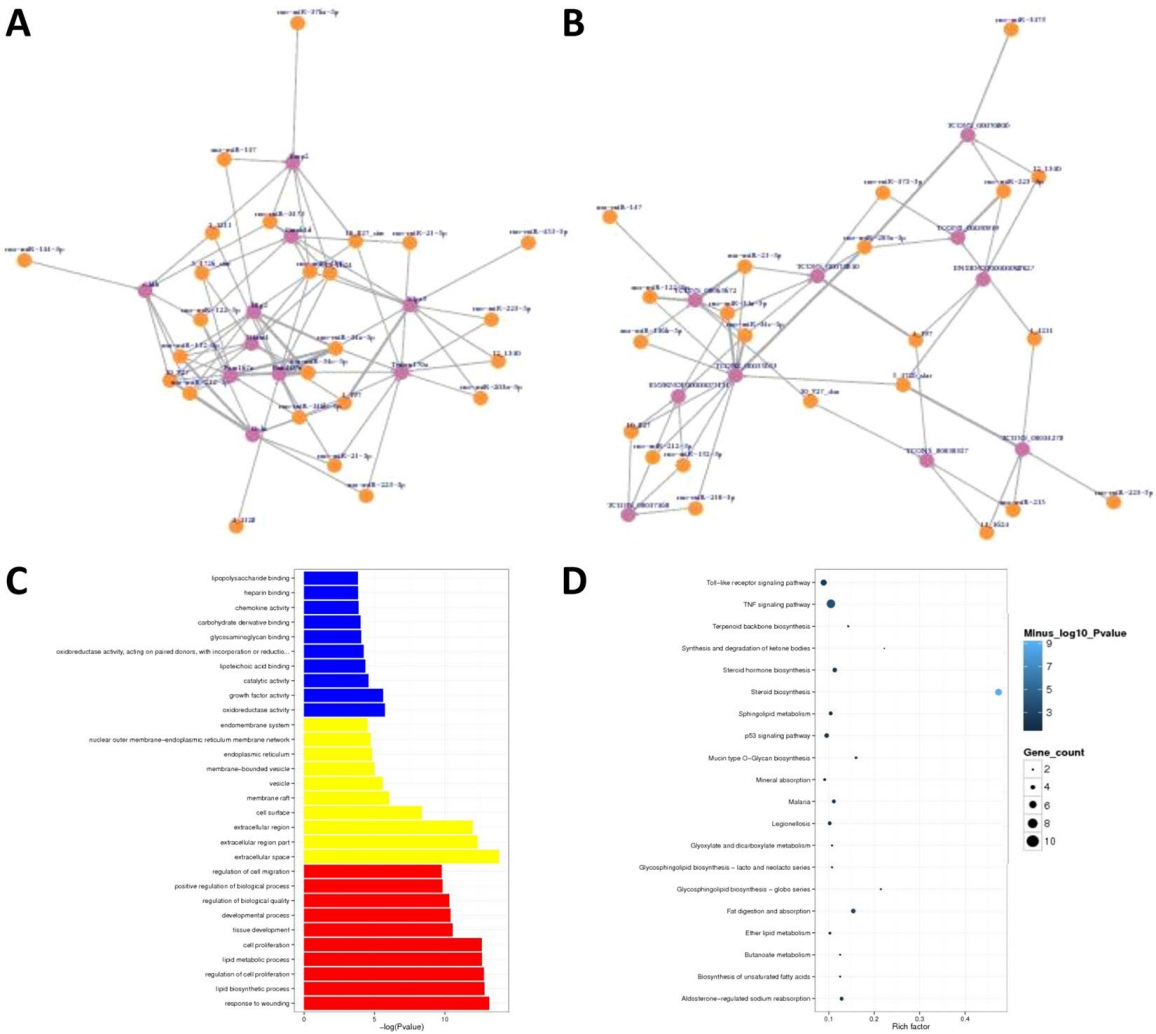
Annotation of differentially expressed miRNAs. (**A**)Network between differentially expressed miRNAs and their potential target genes. (**B**) Co-expression network between differentially expressed miRNAs and lncRNAs. (**C**) Most significantly enriched GO (−log10 (P value)) terms of miRNAs gene symbols according to biological process, marked red; cellular component, marked yellow; molecular function, marked blue. (**D**) KEGG pathway enrichment analysis of up and down regulated miRNAs.

To investigate the possible functions of the differentially expressed miRNAs, we predicted the potential targets of miRNAs in regulatory relationships using Miranda. We constructed a network of significant differentially expressed miRNA transcripts with 10 potential protein-coding genes. Their potential target genes include Emp2, Camk1d, Btg2, Cb1b, Nfam1, Fam167a, Fam107a, Ocln, S1pr3 and Tmem107a (Fig. 4A). We also analyzed the co-expressed lncRNA. There are 10 lncRNA co-expressed with differentially expressed miRNA (Fig. 4B). The ten most enriched GO terms in the domain of:biological process (BP), cellular component (CC) and molecular function (MF) are shown in Fig. 4C. KEGG pathway analysis showed the top 20 pathways that related to the differently expressed miRNAs. As shown in Fig. 4D, the most significant KEGG pathway is the steroid biosynthesis pathway.

### Functional enrichment analysis of differentially expressed circRNAs

**Figure 5:**
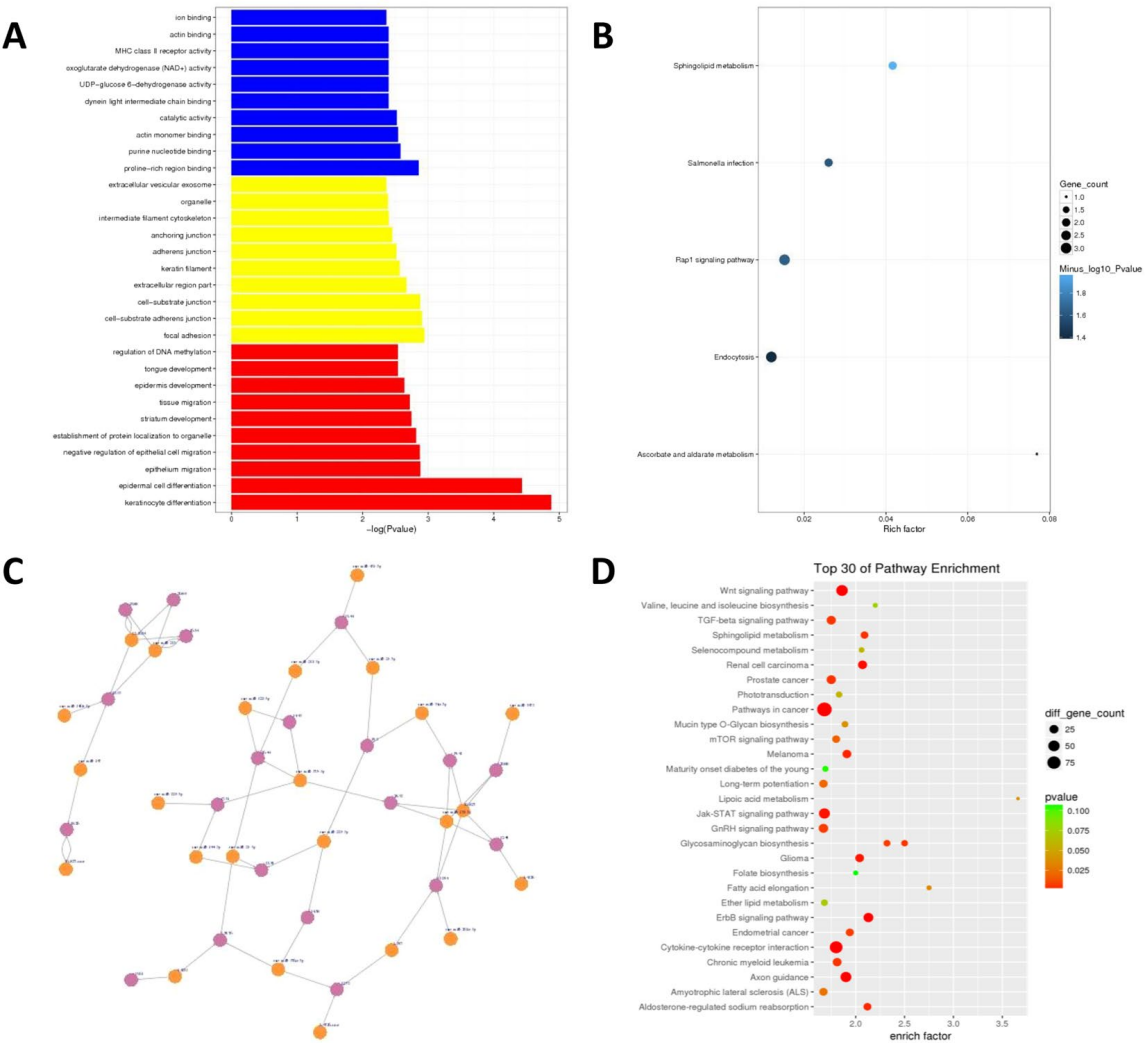
Annotation of differentially expressed circRNAs. (**A**)Most significantly enriched GO (−log10 (P value)) terms of miRNAs gene symbols according to biological process, marked red; cellular component, marked yellow; molecular function, marked blue. (**B**) KEGG pathway enrichment analysis of up and down regulated miRNAs. (**C**) Prediction of interaction networks between differentially expressed miRNAs and circRNAs. (**D**) Annotation of miRNA targets, KEGG pathway enrichment analysis of the mRNA targets of dysregulated miRNAs.

To evaluate how circRNAs are involved in the biological process, cellular components, molecular functions, and pathways, we conducted GO and pathway analyses for the circRNA gene symbols to speculate on the relationship between differentially expressed circRNAs and their potential functions. The 10 most enriched GO terms in each domain are shown in Fig. 5A. In KEGG pathway analysis, sphingolipid metabolism stood out for its high significance level and rich factor (Fig. 5B). To evaluate the potential functions of circRNAs, we investigated potential miRNA binding with circRNAs using Arraystar’s homemade miRNA target prediction software based on TargetScan and miRanda. The results showed that 20 circRNA interacted with 24 miRNAs (Fig. 5C). In addition, with KEGG analysis, we also found that more than 30 proteins in the sphingolipid metabolism are targeted by the differentially expressed cicRNAs or miRNAs (Fig. 5D).

## Discussion

In this study, we investigated and compared the IR-induced changes in the transcription of miRNAs and circRNAs in irradiated esophageal tissues and non-radiated esophageal tissues using RNA-seq. Comparing the transcriptome profiles in response to IR, we identified 27 differentially expressed miRNAs between control and radiation-treated samples. Of the 27 miRNAs, 7 were down-regulated, and the others were up-regulated. There were 197 differentially expressed circRNAs in total, of which 110 were down-regulated and 87 were up-regulated.

We then analyzed the function of the differentially expressed miRNAs and circRNAs. The differentially expressed miRNAs were involved in many cellular processes, such as cell proliferation, cell migration, and lipid metabolism. Of these processes, the most important one is lipid metabolism, particularly steroid metabolism. Previous studies have revealed that lipid metabolism plays roles in the regulation of oncogenic signal pathways^31^. It has been suggested that upregulation of de novo lipogenesis, which accelerates the final steps in de novo synthesis of fatty acids, is one unique feature of cancer cells and is associated with poor prognosis in cancer patients^32^. It is also found that some steroids are enrolled in immune responses^33^ and DNA repair^34^, so that might be how the esophagus responds to ionizing radiation. In particular, the steroid metabolism gene CYP2D polymorphism is associated with clinical late toxicity in patients treated with conformal radiotherapy^34^.

The differentially expressed circRNAs are involved in cellular macromolecule metabolic processes, ion binding, enzyme binding, nucleotide binding and cellular components, the most important of which is sphingolipid metabolism. Sphingolipids are membrane lipids that regulate the fluidity and subdomain structure of lipid bilayers^35^. Evidence has also shown that some species of sphingolipids can function as bio-effector molecules, which are involved in many cancer biology processes, including apoptosis, cell proliferation, cell migration and inflammation^36^. Therefore, sphingolipids play a crucial role in cancer development and progression, and they have some impact on cancer therapy efficacy^37,38^. Our data show that the expression of circRNAs related to sphingolipid metabolism changed significantly during irradiation, which suggests that sphingolipid metabolism might be an important link in radiation-induced esophageal injury. However, we can not indicate that all the sphingolipid disregulations are related to the emergence of anomalous mi/circ RNAs. Sphingolipid metabolism might serve as a drug target for abolishing the side effect of esophageal irradiation.

Evidence has also shown that miRNA plays an important role in many cellular processes. The aim of this study was to analyze RNA-seq data from normal esophageal tissue and irradiated esophageal tissues and used computational approaches to identify and characterize differentially expressed miRNAs and circRNAs. The experimentally validated and literature-based results were inspected, and genes involved in radiation, immune and inflammatory response were pooled^39^. Moreover, our results provide interesting potential clues into the mechanism of ionizing radiation-induced esophageal injury. Since the roles of circRNAs in radiation-induced esophageal injury have not yet been fully identified and understood, this analysis should provide a valuable resource and information for future studies.

## Conclusions

We have identified a substantial number of specific IR-induced miRNAs and circRNAs in radiation-induced esophageal injury. We have shown that there are potential immunological functions for them in the pathogenesis of esophageal injury. Moreover, our results provide interesting potential clues into the mechanism of ionizing radiation-induced esophageal injury. Since the roles of circRNAs in radiation-induced esophageal injury have not yet been fully identified and understood, this analysis should provide a valuable resource and information for future studies.

## Data Availability

The datasets are available from the corresponding author on reasonable request.

## References

[1] Bar-Ad V (2014). Treatment-related acute esophagitis for patients with locoregionally advanced non-small cell lung cancer treated with involved-field radiotherapy and concurrent chemotherapy. Am J Clin Oncol.

[2] Vanagunas A, Jacob P, Olinger E (1990). Radiation-induced esophageal injury: a spectrum from esophagitis to cancer. Am J Gastroenterol.

[3] Zhang Z (2014). Risk factors of radiation-induced acute esophagitis in non-small cell lung cancer patients treated with concomitant chemoradiotherapy. Radiat Oncol.

[4] Stephans KL (2014). Esophageal dose tolerance to hypofractionated stereotactic body radiation therapy: risk factors for late toxicity. Int J Radiat Oncol Biol Phys.

[5] Shimizu T (1990). Radiation-induced esophageal cancer: a case report and a review of the literature. Jpn J Surg.

[6] Levi F (2005). Increased risk of esophageal cancer after breast cancer. Ann Oncol.

[7] Morton LM (2012). Risk of treatment-related esophageal cancer among breast cancer survivors. Ann Oncol.

[8] Zablotska LB (2005). Increased risk of squamous cell esophageal cancer after adjuvant radiation therapy for primary breast cancer. Am J Epidemiol.

[9] Scholl B (2001). Esophageal cancer as second primary tumor after breast cancer radiotherapy. Am J Surg.

[10] Micke O (1999). Radiation-induced esophageal carcinoma 30 years after mediastinal irradiation: case report and review of the literature. Jpn J Clin Oncol.

[11] Hall E. J. & Giaccia, A. J. *Radiobiology for the Radiologist*. Philadelphia, PA, USA: Lippincott Williams & Wilkins (2006).

[12] Criswell T (2003). Transcription factors activated in mammalian cells after clinically relevant doses of ionizing radiation. Oncogene.

[13] Burns TF, El-Deiry WS (2003). Microarray analysis of p53 target gene expression patterns in the spleen and thymus in response to ionizing radiation. Cancer Biol Ther.

[14] Obe G, Johannes C, Schulte-Frohlinde D (1992). DNA double-strand breaks induced by sparsely ionizing radiation and endonucleases as critical lesions for cell death, chromosomal aberrations, mutations and oncogenic transformation. Mutagenesis.

[15] Droge W (2002). Free radicals in the physiological control of cell function. Physiol Rev.

[16] Epperly MW (2001). Modulation of radiation-induced cytokine elevation associated with esophagitis and esophageal stricture by manganese superoxide dismutase-plasmid/liposome (SOD2-PL) gene therapy. Radiat Res.

[17] Patel ZS (2012). Ionizing radiation enhances esophageal epithelial cell migration and invasion through a paracrine mechanism involving stromal-derived hepatocyte growth factor. Radiat Res.

[18] Spriggs KA, Bushell M, Willis AE (2010). Translational regulation of gene expression during conditions of cell stress. Mol Cell.

[19] Simone NL (2009). Ionizing radiation-induced oxidative stress alters miRNA expression. PLoS One.

[20] Hallahan DE (1989). Increased tumor necrosis factor alpha mRNA after cellular exposure to ionizing radiation. Proc Natl Acad Science USA.

[21] Chen LL, Yang L (2015). Regulation of circRNA biogenesis. RNA Biol.

[22] Li L (2017). Comprehensive CircRNA expression profile and selection of key CircRNAs during priming phase of rat liver regeneration. BMC Genomics.

[23] Chen J (2017). Circular RNA WDR77 target FGF-2 to regulate vascular smooth muscle cells proliferation and migration by sponging miR-124. Biochem Biophys Res Commun.

[24] Zhou B, Yu JW (2017). A novel identified circular RNA, circRNA_010567, promotes myocardial fibrosis via suppressing miR-141 by targeting TGF-beta1. Biochem Biophys Res Commun.

[25] Xu XW (2017). Circular RNA hsa_circ_000984 promotes colon cancer growth and metastasis by sponging miR-106b. Oncotarget.

[26] Gao YL (2017). Circular RNA expression profiles reveal that hsa_circ_0018289 is up-regulated in cervical cancer and promotes the tumorigenesis. Oncotarget.

[27] Zhong Z (2017). Circular RNA MYLK as a competing endogenous RNA promotes bladder cancer progression through modulating VEGFA/VEGFR2 signaling pathway. Cancer Lett.

[28] Yang, Y. *et al*. Novel Role of FBXW7 Circular RNA in Repressing Glioma Tumorigenesis. *J Natl Cancer Inst* **110**(**3**) (2018). 10.1093/jnci/djx166 PMC6019044 28903484

[29] Salmena L (2011). A ceRNA hypothesis: the Rosetta Stone of a hidden RNA language?. Cell.

[30] Wang Z, Gerstein M, Snyder M (2009). RNA-Seq: a revolutionary tool for transcriptomics. Nat Rev Genet.

[31] Grunt, T. W. Interacting Cancer Machineries: Cell Signaling, Lipid Metabolism, and Epigenetics. *Trends Endocrinol Metab* (2017). 10.1016/j.tem.2017.11.003 29203141

[32] Menendez JA, Lupu R (2007). Fatty acid synthase and the lipogenic phenotype in cancer pathogenesis. Nat Rev Cancer.

[33] Hafner LM, Cunningham K, Beagley KW (2013). Ovarian steroid hormones: effects on immune responses and Chlamydia trachomatis infections of the female genital tract. Mucosal Immunology..

[34] Damaraju S (2006). Association of DNA repair and steroid metabolism gene polymorphisms with clinical late toxicity in patients treated with conformal radiotherapy for prostate cancer. Clin. Cancer Res.

[35] Futerman AH, Hannun YA (2004). The complex life of simple sphingolipids. EMBO Report.

[36] Maceyka M, Spiegel S (2014). Sphingolipid metabolites in inflammatory disease. Nature.

[37] Kok JW, Sietsma H (2004). Sphingolipid metabolism enzymes as targets for anticancer therapy. Curr Drug Targets.

[38] Modrak DE, Gold DV, Goldenberg DM (2006). Sphingolipid targets in cancer therapy. Mol Cancer Ther.

[39] Georgakilas AG (2015). Emerging molecular networks common in ionizing radiation, immune and inflammatory responses by employing bioinformatics approaches. Cancer Lett.

